# Can Gene Expression Differentiate Patients With Heart Failure due to Coronary Heart Disease From Patients With Coronary Disease Without Heart Failure?

**DOI:** 10.1111/jcmm.70727

**Published:** 2025-09-03

**Authors:** Józefa Dąbek, Joanna Głogowska‐Ligus, Mieczysław Piechota

**Affiliations:** ^1^ Department of Cardiology, Faculty of Health Sciences in Katowice Medical University of Silesia Katowice Poland; ^2^ Department of Epidemiology, Faculty of Public Health in Bytom Medical University of Silesia Katowice Poland; ^3^ Department of Cardiology Municipal Hospital in Tychy Tychy Poland

**Keywords:** heart failure, risk factors, transforming growth factor β1

## Abstract

The aim of the study was to analyse and compare the transcriptional activity of the TGF‐β1 gene and its receptors in patients with heart failure based on coronary artery disease and patients with coronary artery disease without heart failure, taking into account the presence of individual risk factors, the stage of heart failure and the number of diseased coronary arteries. The study included 105 patients with advanced heart failure (NYHA III–IV) and 52 patients with coronary artery disease without heart failure. The study was performed using the QRT‐PCR technique. The transcriptional activity of the TGF‐β1 gene and its type III receptor was significantly lower in patients with advanced heart failure. In this group of patients with risk factors such as previous myocardial infarction, family history, overweight/obesity and arterial hypertension, a statistically significant reduction in TGF‐β1 gene expression was demonstrated. In patients with heart failure, changes in the transcriptional activity of all receptor's genes were found when these patients were burdened with risk factors. The reduced transcriptional activity of TGF‐β1 and its type III receptor demonstrated in patients with heart failure may be a useful marker in clinical practice for assessing the progression of coronary artery disease towards the development of heart failure, as well as its decompensation and compensation.

## 1 Introduction

Epidemiological data from various studies, despite some differences, agree that the number of patients with heart failure is constantly increasing. The scale of the problem is evidenced by the forecasts of the American Heart Association (AHA), according to which the number of hospitalisations due to heart failure (HF) by 2030 will increase by 25% [1].

In HF, the expression of many genes is altered with their activation and inactivation, similar to the condition observed in the fetal period. Changes in the activity of genes encoding sarcomere proteins, intracellular calcium circulation, and proteins involved in cardiac metabolism are particularly important. Various types of pathological stimuli can activate cardiomyocyte hypertrophy pathways through:

- Mechanical stretching directly activating intracellular signal transduction pathways;
- Mechanical stretching to stimulate the release of local factors;
- Neurohumoral pathways causing the release of paracrine and/or endocrine factors.

The mechanism by which mechanical tension is converted into biological signals is not fully understood. There are two hypotheses:

- Mechanical stretching deforms the sarcolemma causing conformational changes in transmembrane proteins and those associated with cell membranes (phospholipases, protein kinases C, tyrosine kinases and ion channels);
- Mechanical stretching modulates the interactions between the extracellular matrix (ECM) and the integrins binding it to the cytoskeleton and intracellular messenger molecules (adhesion kinase).

Neurohormonal pathways have been studied in more detail. The extracellular matrix is a reservoir of numerous growth factors released as a result of its degradation. One of such factors is transforming growth factor β (TGF‐β) associated with decorin that maintains tissue integrity through interaction with fibronectin and thrombospondin [2, 3]. Moreover, the mentioned factor is an important protein influencing changes in heart cells in the course of heart failure [4].

TGF‐β is a member of the superfamily of peptide messenger molecules that regulate cell proliferation and differentiation. It participates in physiological and pathological processes such as growth, cell differentiation, inflammation, neoplasm, atherosclerosis, angiogenesis, tissue repair processes and fibrosis [5, 6, 7]. TGF‐β is a superfamily of proteins that includes five isoforms: TGF‐β1, TGF‐β2 and TGF‐β3, and bone morphogenetic proteins (BMP). The isoforms have almost 70% amino acid sequence homology. Their high similarity also includes comparable activity, activation of the same receptors and signalling pathways, and similar biological effects [8, 9, 10]. The TGF‐β receptor system includes two membrane receptors: the type I receptor (TGF‐β RI) and the type II receptor (TGF‐β RII), which are stimulated after ligand binding to the receptor III (TGF‐β RIII), which is only indirectly involved in signal transduction [11].

Studies conducted so far have shown that TGF‐β transcription and its subsequent secretion occur in cells of the immune system, including peripheral blood mononuclear cells (PBMC) [12]. These cells have a proven negative impact on the remodelling of the heart muscle in its failure [13], and previously published studies confirm their usefulness in analyses using the determination of gene transcriptional activity in patients affected by various diseases [14, 15]. Taking the above information into account, PBMCs become an interesting and easily accessible cell population with potential importance in monitoring the course and assessing the progression of heart failure.

Therefore, the aim of this study was to analyse the transcriptional activity of the TGF‐β1 gene and its receptors in patients with underlying coronary artery disease and heart failure and in patients with coronary artery disease without heart failure, taking into account the presence of risk factors, the severity of heart failure, and the number of obstructed coronary arteries.

## 2 Materials and Methods

### 2.1 Study Group

The research was commenced after obtaining the consent of the Bioethics Committee of the Medical University of Silesia in Katowice (KNW/0022/KB1/1/17/I/12). The study group included all patients consecutively admitted to the Cardiology Department of the Municipal Hospital in Tychy (Poland), due to decompensated heart failure in NYHA class III and IV arising in the course of advanced coronary artery disease. The criteria for inclusion into the study were: age over 18 years written informed consent to participate in the study, documented coronary artery disease (significant and critical stenosis changes and closed coronary arteries in coronary angiography, condition after coronary artery plastic surgery or after coronary artery bypass surgery, and transthoracic echocardiography), clinically diagnosed acute decompensated heart failure in NYHA functional class III‐IV. The exclusion criteria included: no consent of the patient to participate in the study, no confirmation of coronary artery disease, heart failure caused by other causes, for example, defects, shortness of breath caused by diseases of other organs: lungs, liver, kidneys, cancer, difficult contact: post‐stroke condition, mental disorders.

A total of 157 (100%) people were qualified for the study group, including 105 (66.88%) patients with advanced heart failure in the course of coronary artery disease and 52 (33.12%) patients with a coronary artery disease without heart failure, who were in the control group. The patients enrolled in the study were assessed in the first 24 h after admission to the hospital, and then after 4–8 weeks during the correction of heart failure. The material for molecular tests was peripheral blood collected from the antecubital vein of patients 1 day after admission to the Department of Cardiology, and then after 4–8 weeks.

### 2.2 Molecular Analysis

The template for determining the number of copies of selected mRNA fragments of the studied genes in the *real‐time* quantitative reverse transcription polymerase chain reaction *PCR* (QRT‐PCR) was the RNA obtained during the extraction. A modified Chomczyński and Sacchi method was used for ribonucleic acid extraction using the TRIzol Reagent (Invitrogen) [16]. RNA integrity was assessed in a 1% agarose gel with ethidium bromide (0.5 mg/mL) by electrophoresis followed by UV transillumination (λ = 260 nm). The quantitative analysis of mRNA was performed using the QuantiTect SYBR Green reverse‐transcription polymerase chain reaction (RT‐PCR) Master Mix kit in the reaction mixture with the composition and reaction conditions recommended by the manufacturer (Qiagen). The number of mRNA molecules for the studied genes was determined based on the kinetics of the QRT‐PCR reaction using the Engine Opticon deoxyribonucleic acid (DNA) sequence detector (MJ Research) and the kit containing the fluorescent SYBR‐Green I dye with a maximum absorbance at 497 nm. Based on the plotted standard curve, the number of DNA copies of the analysed genes was determined. Moreover, the reliability was controlled by standard curves (5‐points with 10‐fold dilutions for every new primer pair) and two reference genes were used in this study—β‐actin and glyceraldehyde‐3‐phosphate dehydrogenase (GAPDH). Median absolute deviation scaling was used for normalisation.

The sequences of oligonucleotide primers for the quantitative QRT‐PCR reaction were designed based on the PrimerExpressTM Version 1.0 computer program, based on the sequences of the test genes from the GenBank database http://www.ncbi.nlm.nih.gov/genbank.

### 2.3 Statistical Analysis

The obtained data were collected in an Excel spreadsheet and exported to the Med Calc program. Mean values ($\bar{x}$) and standard deviation (SD) were calculated. The normality of the distribution of the examined parameters was checked with the Kolmogorov‐Smirnows test. As the parameters tested did not show any signs of normality, the non‐parametric U Mann–Whitney and Wilcoxon tests were used in the statistical analysis. A significant level of *p* < 0.05 was adopted as statistically significant.

## 3 Results

The general characteristics of the patients qualified for the study group, taking into account the risk factors for coronary atherosclerosis, were presented in Table 1.

**TABLE 1:** General characteristics of the patients with advanced heart failure in the course of coronary artery disease and patients with coronary artery disease without heart failure, taking into account the risk factors of coronary atherosclerosis.

Variables	Studied group (A + C) *n* = 157		Patients with HF in the course of CAD (A) *n* = 105		Patients with CAD without HF (C) *n* = 52	
Age ($\bar{x}$ ± SD) (years)	68.74 ± 9.98		71.14 ± 9.18		63.9 ± 9.8	
Number of studied patients (*n*; %)	*n*	%	*n*	%	*n*	%
Total number of studied patients	157	100	105	66.9	52	33.1
Number of patients in a given group	157	100	105	100	52	100
Sex						
Female	4	2.5	2	2	2	3.8
Male	153	97.5	103	98	50	96.2
Body weight (based on BMI results)						
Normal weigh	38	24.2	28	26.6	10	19.3
Overweight	57	36.6	30	28.6	27	51.9
Obesity	62	39.5	47	44.8	15	28.8
Arterial hypertension	153	97.5	101	96.2	52	100
Diabetes	65	41.4	47	44.8	18	34.6
Tobacco smoking	59	37.6	44	41.9	15	28.8
Burdensome family history	92	58.6	87	82.9	5	9.6
Total cholesterol > 200 mg%	30	19.1	24	22.6	6	11.5

Abbreviations: BMI, Body Mass Index; CAD, coronary artery disease; HF, heart failure; *n*, number of studied patients; SD, standard deviation; $\bar{x}$, mean value.

Characteristics of the study group takin into account results of differences test between values of TGF‐β1 gene and its receptors transcriptional activity of patients with advanced heart failure in the course of coronary artery disease and occurrence of individual risk factors was presented in Table 2.

**TABLE 2:** Characteristics of the study group taking into account results of the differences test between values of TGF‐β1 gene and its receptors transcriptional activity of patients with advanced heart failure in the course of coronary artery disease and occurrence of individual risk factors.

Risk factors	TGF‐β1	TGF‐β1 RI	TGF‐β1 RII	TGF‐β1 RIII
	*p*	*p*	*p*	*p*
Hypertension	*p* < 0.05	NS	NS	*p* < 0.05
Diabetes	NS	NS	NS	*p* < 0.05
Body mass	*p* < 0.05	NS	NS	*p* < 0.05
History of myocardial infarction	*p* < 0.05	NS	NS	*p* < 0.05
Positive family history	*p* < 0.05	*p* < 0.05	*p* < 0.05	*p* < 0.05
Atherosclerosis in the CNS	NS	*p* < 0.05	*p* < 0.05	*p* < 0.05
Carotid atherosclerosis	NS	*p* < 0.05	NS	*p* < 0.05
Generalised atherosclerosis	NS	NS	*p* < 0.05	*p* < 0.05
Peripheral atherosclerosis	NS	NS	NS	*p* < 0.05
Tobacco smoking	NS	NS	NS	NS
TC	NS	NS	NS	*p* < 0.05
HDL	NS	NS	NS	*p* < 0.05
LDL	NS	NS	NS	*p* < 0.05
TG	NS	NS	NS	*p* < 0.05

Abbreviations: CNS, central nervous system; HDL, high‐density lipoprotein; LDL, low‐density lipoprotein; NS, statistically insignificant result; RI, receptor 1 for TGF‐β1; RII, receptor 2 for TGF‐β1; RIII, receptor 3 for TGF‐β1; TC, total cholesterol; TGF‐β1, transforming growth factor β1; TG, triglycerides.

The conducted analysis showed that the risk factors influencing the change of transcriptional activity of the studied genes include: a history of myocardial infarction (TGF‐β1 and TGF‐β1R III), negative family history (TGF‐β1 and TGF‐β1 RI, TGF‐ β1 RII, TGF‐β1 RIII), overweight/obesity (TGF‐β1 and TGF‐β1 RIII), hypertension (TGF‐β1 and TGF‐β1 RIII), atherosclerosis (TGF‐β1 RI, TGF‐β1 RII, TGF‐β1 RIII), diabetes and lipid disorders (TGF‐β1 RIII).

Characteristics of the study group taking into account results of differences test between values of TGF‐β1 gene and its receptors transcriptional activity of patients with advanced heart failure in the course of coronary artery disease and left ventricular ejection fraction was presented in Table 3.

**TABLE 3:** Characteristics of the studied group of patients with advanced heart failure in the course of coronary artery disease, taking into account the transcriptional activity of the studied genes and the left ventricular ejection fraction.

Examined parameter	EF%	EF% > 35 (*n* = 52)	EF% < 35 (*n* = 53)
TGF‐β1	EF% > 35	—	NS
	EF% < 35	NS	—
TGF‐β1 RI	EF% > 35	—	NS
	EF% < 35	NS	—
TGF‐β1 RII	EF% > 35	—	*p* < 0.05
	EF% < 35	*p* < 0.05	—
TGF‐β1 RIII	EF% > 35	—	NS
	EF% < 35	NS	—

Abbreviations: EF, left ventricular ejection fraction; GAPDH, Glyceraldehyde‐3‐phosphate dehydrogenase; NS, a statistically insignificant result; RIII, receptor 3 for TGF‐β1; RII, receptor 2 for TGF‐β1; RI, receptor 1 for TGF‐β1; TGF‐β1, transforming growth factor β1.

A significant reduction in the transcriptional activity of the constitutive gene of the type II TGF‐β receptor has been demonstrated in patients with a low ejection fraction.

Characteristics of the study group of patients with advanced heart failure based on coronary artery disease taking into account results of differences tests between values of TGF‐β1 gene and its receptors transcriptional activity and number of diseased coronary arteries was presented in Table 4. In Table 5 characteristics of the study group of patients with coronary artery disease without heart failure taking into account results of differences test between values of TGF‐β1 gene and its receptors transcriptional activity and number of diseased coronary arteries was presented.

**TABLE 4:** Characteristics of the studied group of patients with advanced heart failure in the course of coronary artery disease taking into account the transcriptional activity of the investigated genes and the number of diseased coronary arteries.

Examined parameter	Coronary artery disease	Single vessel (*n* = 19)	Two vessels (*n* = 14)	Multivessel (*n* = 19)
TGF‐β1	Single vessel	—	*p* < 0.05	*p* < 0.05
	Two vessels	*p* < 0.05	—	NS
	Multivessel	*p* < 0.05	NS	—
TGF‐β RI	Single vessel	—	NS	NS
	Two vessels	NS	—	NS
	Multivessel	NS	NS	—
TGF‐β RII	Single vessel	—	NS	NS
	Two vessels	NS	—	NS
	Multivessel	NS	NS	—
TGF‐β RIII	Single vessel	—	*p* < 0.05	*p* < 0.05
	Two vessels	*p* < 0.05	—	NS
	Multivessel	*p* < 0.05	NS	—

Abbreviations: NS, a statistically insignificant result; RI, receptor 1 for TGF‐β1; RII, receptor 2 for TGF‐β1; RIII, receptor 3 for TGF‐β1; TGF‐β1, transforming growth factor β1.

**TABLE 5:** Characteristics of the studied group of patients with coronary artery disease without heart failure taking into account the transcriptional activity of the studied genes and the number of diseased coronary arteries.

Examined parameter	Coronary artery disease	Single vessel (*n* = 4)	Two vessels (*n* = 7)	Multivessel (*n* = 12)
TGF‐β1	Single vessel	—	*p* < 0.05	*p* < 0.05
	Two vessels	*p* < 0.05	—	NS
	Multivessel	*p* < 0.05	NS	—
TGF‐β RI	Single vessel	—	NS	NS
	Two vessels	NS	—	NS
	Multivessel	NS	NS	—
TGF‐β RII	Single vessel	—	NS	NS
	Two vessels	NS	—	NS
	Multivessel	NS	NS	—
TGF‐β RIII	Single vessel	—	NS	NS
	Two vessels	NS	—	NS
	Multivessel	NS	NS	—

Abbreviations: NS, a statistically insignificant result; RI, receptor 1 for TGF‐β1; RII, receptor 2 for TGF‐β1; RIII, receptor 3 for TGF‐β1; TGF‐β1, transforming growth factor β1.

In patients with advanced heart failure in the course of coronary artery disease, a statistically significant decrease in the activity of the cytokine gene and its receptor III was observed along with the severity of coronary artery disease (in two‐ and multi‐vessel disease).

Analysing the transcriptional activity of TGF‐β1 genes and its receptors and the number of diseased coronary arteries among patients with coronary artery disease without heart failure, a statistically significant decrease in the activity of the cytokine gene was noted, along with the severity of coronary artery disease (in two‐ and multivessel coronary artery disease).

**FIGURE 1:**
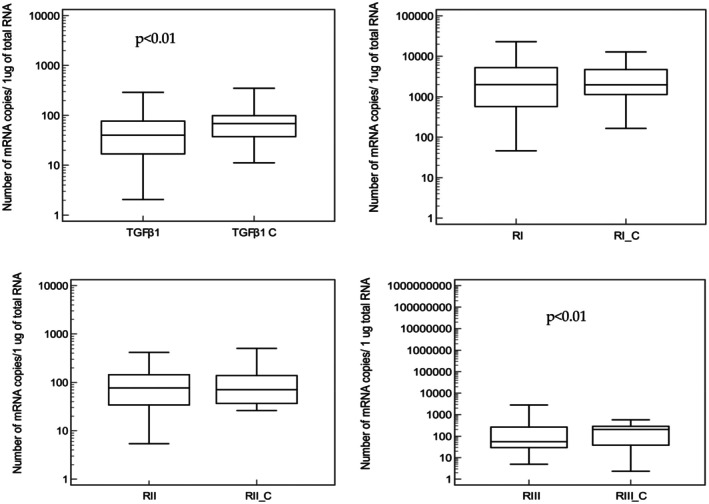
Transcriptional activity of the TGF‐β1 gene and its receptors in patients with advanced heart failure in the course of coronary artery disease and patients with coronary artery disease without failure (C). RIII, receptor 3 for TGF‐β1; RII, receptor 2 for TGF‐β1; RI, receptor 1 for TGF‐β1; TGF‐β1, transforming growth factor β1. No additional markings at the end of the abbreviation apply to patients with advanced heart failure in the course of coronary artery disease, and the additional mark—‘C’ refers to the group of patients with coronary artery disease without heart failure.

The transcriptional activity of the TGF‐β1 gene and its receptors in the studied group of patients with advanced heart failure in the course of coronary artery disease and patients with coronary artery disease without heart failure caused by coronary artery disease was presented in Figure 1.

The transcriptional activity of the TGF‐β1 gene and its receptor III was statistically significantly lower in the group of patients with advanced heart failure in the course of coronary artery disease, compared to the control group.

## 4 Discussion

Results of experimental population trials on heart failure are difficult to compare. As observed by Niederseer et al., patients recruited for clinical trials only partially represent those encountered in everyday practice. In order for data from clinical trials to be implemented in everyday practice, numerous experimental analyses are needed [17]. Previously conducted studies with the use of oligonucleotide microarrays emphasise the role of Transforming Growth Factor β1 (TGF‐β) in cardiovascular diseases [18].

Mentioned particle is present in all leukocyte‐derived cell lines: and its expression causes both auto‐ and paracrine actions that control the differentiation, proliferation, and activity status of immune cells [19]. The effect of the aforementioned cytokine on damage‐induced leukocytes depends on its concentration and the degree of cell maturity. Transforming growth factor β1 may stimulate or inhibit it [20].

It has to be emphasised that TGF‐β can play either an atheroprotective or atherogenic role [21]. It can induce fibrosis in various cells, including fibroblasts in the heart [22]. But by inhibiting the expression of adhesion molecules, increasing the expression of structural proteins of the extracellular matrix, and inhibiting metalloproteinases responsible for its degradation, TGF‐β also shows anti‐atherosclerotic effects [23, 24]. As a result of the mentioned actions, TGF‐β contributes to the maintenance of the proper condition of the vessels, and in the case of the development of atherosclerotic plaques, it stabilises them. The presented profile of TGF‐β activity, in the light of experimental studies, allows it to be classified as a cytokine with a protective effect on vessels and stabilising atherosclerotic plaques as a result of strengthening the fibrous cap, and the decrease in its production or expression may be considered one of the factors destabilising it [25].

Wang et al. demonstrated that total cholesterol and LDL cholesterol inhibited TGF‐β induced signalling pathways, leading to the blockade of cardioprotective mechanisms [26]. Similar results were obtained by Chen et al. [27]. Dąbek et al. showed a decrease in TGF‐β1 expression in patients with acute coronary syndrome [28]. Law et al. revealed a significant role of TGF‐β in the processes of cardiac fibrosis caused by the toxic effects of alcohol [29].

Also, diabetes and obesity, by stimulating TGF‐β, affect the progression of myocardial fibrosis, which was confirmed by Cavalera et al. and Bugyei‐Twum et al. [30, 31]. These results were also confirmed by Gramley et al. [32].

In the present study, a significant decrease in the transcriptional activity of the TGF‐β1 gene was found in patients with decompensation of heart failure. On the other hand, the same group, in the period of clinical correction of cardiac insufficiency, already showed an increase in the expression of the tested cytokine, although it was statistically significantly lower than that found in the control group. Such a result may suggest that a significant decrease in the transcriptional activity of TGF‐β1 may contribute to the decompensation of heart failure, while significantly higher expression in these patients during the stable period confirms the protective role of the high activity of the described cytokine. Moreover, significantly lower levels of the described cytokine among patients with heart failure compared to patients without it may indicate its participation in the development of heart failure, which was also found by Basma et al. in their study [33].

In our study, among patients with decompensated and compensated heart failure in the course of coronary artery disease, a significantly reduced expression of the gene for the TGF‐β receptor III was also found. It was observed that during the correction of heart failure, the expression of the gene for the TGF‐β receptor III was increased compared to the transcriptional activity of the discussed cytokine in the decompensation stage, but still significantly lower than in the control group. As shown by Chu et al., the TGF‐β type III receptor inhibits the signalling pathways of transforming growth factor β1, directly neutralising TGF‐β1 and indirectly preventing the formation of the active TGF‐β RI/TGF‐β RII complex [34]. In the conducted studies, the inhibitory effect of the type III TGF‐β receptor on the process of myocardial fibrosis was also demonstrated by Hermida et al. [35]. Liang et al., looking for other mechanisms influencing the process of myocardial fibrosis, showed a relationship between the concentration of microRNA‐21 and the type III receptor for TGF‐β, revealing another new factor regulating the process of fibrosis. The increase in TGF‐β RIII expression may inhibit the expression of microRNA‐21, thus blocking the progress of fibrosis [36].

In addition, significant changes in the transcriptional activity of the studied genes were observed in the study, in terms of risk factors (atherosclerosis of the central nervous system and peripheral arteries, burdening family history, past myocardial infarction, overweight/obesity, arterial hypertension, diabetes and lipid disorders), heart failure (low ejection fraction) and number of diseased coronary arteries (single‐, two‐ or multi‐vessels) suggest their significant participation in processes related to heart remodelling. According to Lelonek et al., the number of deaths in Poland due to heart failure reaches almost 150 thousand annually. As an important cause of high rates of mortality, not only is a large number of patients with heart failure indicated, but also the serious prognosis of patients over 60 years of age, especially in the 75+ population. In Poland, only 57% of patients survive 5 years from the moment of diagnosis of HF, while in the age group 75+, half of patients survive not about 4 years [37].

Diabetes remains an important problem in modern societies, present in as many as 44.8% of respondents with heart failure and 34.6% of patients without heart failure. Diabetic patients are at the highest risk of developing complications of atherosclerosis, which is confirmed by studies inter alia, by Elendu et al. which indicate a relationship between diabetes and the severity of heart failure [38]. Based on data from Palazzuoli and Iacoviello, study patients with T2DM have a 30% greater risk of requiring hospitalisation for acute HF than do patients without T2DM [39]. In the results obtained in patients with advanced heart failure in the course of coronary artery disease, compared with the results of patients with coronary artery disease without heart failure, who constituted the control group, more frequent severe family history was found (82.9% vs. 9.6%).

The study included patients with decompensated heart failure, whose mean left ventricular ejection fraction was 32%, and patients with coronary heart disease without heart failure with an ejection fraction of 55%. A follow‐up study of patients with HF, performed 4–8 weeks after the first study, showed a slight increase in the ejection fraction, up to 35%. As suggested by the literature data, patients with an ejection fraction below 35% constitute a group of patients, particularly at risk of sudden cardiac deaths and frequent hospitalisations.

The limitations of the study include the small number of respondents included in the study and the lack of equality of the compared groups. Based on extensive research on the molecular basis of heart failure in the course of coronary artery disease, many mechanisms have been identified leading to damage of the heart muscle as a result of hypoxia and infarction. Due to the numerous dependencies and interrelationships between the signalling pathways of genes involved in the repair processes presented above, it is difficult to give an unambiguous answer to the question of why heart failure develops. Therefore, it is necessary to continue research work aimed at elucidating these complex mechanisms and then implementing appropriate therapeutic measures aimed at preventing the formation and development of heart failure.

To conclude, obtained results and to sum up the discussion, it has to be said that a statistically significant reduction in the transcriptional activity of the TGF‐β1 gene and its III receptor in the group of patients with advanced heart failure and their further reduction in the decompensation stage suggests the significant importance of these genes in the development and decompensation of heart failure, making them useful in clinical practice as diagnostic and prognostic markers, identifying people with coronary artery disease and a high risk of developing heart failure and its decompensation and compensation. Moreover, the changes in the transcriptional activity of TGF‐β1 genes and its receptors in the studied group of patients with advanced heart failure in the course of coronary disease under the influence of some risk factors of reduced left ventricular ejection fraction suggest their significant role in the development and progression of heart failure. Lastly, the importance of risk factors in the development, advancement, and decompensation of heart failure, confirmed by the transcriptional activity of the studied genes, enables early implementation of the principles of prevention and intensive non‐pharmacological (with risk factors elimination) and pharmacological treatment to improve the quality of life and health of the studied group of patients.

## Author Contributions

**Józefa Dąbek:** conceptualization (lead), data curation (equal), formal analysis (equal), investigation (lead), methodology (lead), project administration (lead), resources (lead), software (equal), supervision (lead), validation (lead), visualization (equal), writing – original draft (lead), writing – review and editing (lead). **Joanna Głogowska‐Ligus:** data curation (equal), formal analysis (lead), methodology (equal), software (equal), validation (equal), visualization (equal), writing – original draft (equal), writing – review and editing (equal). **Mieczysław Piechota:** conceptualization (supporting), data curation (equal), formal analysis (supporting), investigation (equal), project administration (supporting), resources (supporting), software (supporting), validation (equal), visualization (supporting), writing – original draft (equal), writing – review and editing (equal).

## Ethics Statement

The research was commenced after obtaining the consent of the Bioethics Committee of the Medical University of Silesia in Katowice (KNW/0022/KB1/1/17/I/12) and was conducted taking into account all ethical aspects of experimental study conduct.

## Consent

Informed consent was obtained from all subjects involved in the study.

## Conflicts of Interest

The authors declare no conflicts of interest.

## Data Availability

The data that support the findings of this study are available from the corresponding author upon reasonable request.
